# Effects of short-term cannabidiol treatment on response to social stress in subjects at clinical high risk of developing psychosis

**DOI:** 10.1007/s00213-019-05442-6

**Published:** 2020-01-08

**Authors:** E. Appiah-Kusi, N. Petros, R. Wilson, M. Colizzi, M. G. Bossong, L. Valmaggia, V. Mondelli, P. McGuire, S. Bhattacharyya

**Affiliations:** 1 Department of Psychosis Studies, Institute of Psychiatry, Psychology & Neuroscience (IoPPN), King’s College London, PO Box 63, De Crespigny Park, Denmark Hill, London, SE5 8AF UK; 2 Department of Neurosciences, Biomedicine and Movement Sciences, Section of Psychiatry, Policlinico “G. B. Rossi”, University of Verona, P.le L.A. Scuro 10, 37134 Verona, Italy; 3 Department of Psychiatry, University Medical Centre Utrecht Brain Centre, Utrecht University Utrecht, The Netherlands; 4 Department of Psychology, IoPPN, King’s College London, London, PO Box 77 UK; 5 National Institute for Health Research, Biomedical Research Centre, London, UK; 6 Department of Psychological Medicine, IoPPN, King’s College London, London, UK

**Keywords:** Cannabidiol, Trier Social Stress Test, Ultra-high risk, Psychosis

## Abstract

**Rationale:**

Stress is a risk factor for psychosis and treatments which mitigate its harmful effects are needed. Cannabidiol (CBD) has antipsychotic and anxiolytic effects.

**Objectives:**

We investigated whether CBD would normalise the neuroendocrine and anxiety responses to stress in clinical high risk for psychosis (CHR) patients.

**Methods:**

Thirty-two CHR patients and 26 healthy controls (HC) took part in the Trier Social Stress Test (TSST) and their serum cortisol, anxiety and stress associated with public speaking were estimated. Half of the CHR participants were on 600 mg/day of CBD (CHR-CBD) and half were on placebo (CHR-P) for 1 week.

**Results:**

One-way analysis of variance (ANOVA) revealed a significant effect of group (HC, CHR-P, CHR-CBD (*p* = .005) on cortisol reactivity as well as a significant (*p* = .003) linear decrease. The change in cortisol associated with experimental stress exposure was greatest in HC controls and least in CHR-P patients, with CHR-CBD patients exhibiting an intermediate response. Planned contrasts revealed that the cortisol reactivity was significantly different in HC compared with CHR-P (*p* = .003), and in HC compared with CHR-CBD (*p* = .014), but was not different between CHR-P and CHR-CBD (*p* = .70). Across the participant groups (CHR-P, CHR-CBD and HC), changes in anxiety and experience of public speaking stress (all *p*’s < .02) were greatest in the CHR-P and least in the HC, with CHR-CBD participants demonstrating an intermediate level of change.

**Conclusions:**

Our findings show that it is worthwhile to design further well powered studies which investigate whether CBD may be used to affect cortisol response in clinical high risk for psychosis patients and any effect this may have on symptoms.

## Introduction

Stress plays a major role in the onset and maintenance of psychosis (Pruessner et al. 2017). Exposure to stress in early (Beards et al. 2013) and adult (van Winkel et al. 2008) life has been linked to an increased risk for psychosis. The hypothalamic-pituitary-adrenal (HPA) axis is a key neuroendocrine regulatory system mediating the biological response to stress.

Accumulating evidence suggests that a dysfunction in the HPA-axis might underlie the psychosis continuum (Pruessner et al. 2017). A recent review of the evidence (Appiah-Kusi et al. 2016) suggests that in response to a stressor, patients with established psychosis and those at clinical high risk (CHR) for psychosis (Day et al. 2014; Pruessner et al. 2013) tend to exhibit a blunted cortisol response to both social stress and awakening. Similarly, evidence has shown that individuals at CHR of psychosis also have an impaired psychological response to stress. Another study also found that impaired tolerance to stress was more predictive of transition to psychosis than attenuated psychotic symptoms (Yung et al. 2005), showing that the psychological response to stress is an important predictor for psychosis. While atypical antipsychotics have been shown to reduce elevated diurnal cortisol levels (Zhang et al. 2005; Cohrs et al. 2006; Mondelli et al. 2010), antipsychotic treatment has not been shown to correct the blunted cortisol response observed in psychosis (Mondelli et al. 2010).

The harmful effects of cannabis have been mainly attributed to the effects of its main psychoactive ingredient, delta-9-tetrahydrocannabinol (THC), which has been shown under experimental conditions to induce psychotic and anxiety symptoms in healthy individuals (D’Souza et al. 2005) and exacerbate psychotic symptoms in patients with pre-existing psychosis (D’Souza et al. 2005). In contrast, cannabidiol (CBD), is a safe and well-tolerated (Bergamaschi et al. 2011a) constituent of cannabis and has antipsychotic (Iseger and Bossong 2015; Leweke et al. 2012; McGuire et al. 2017; Bhattacharyya et al. 2010) and anxiolytic (Bergamaschi et al. 2011b) properties. Importantly, preclinical evidence suggests that CBD may attenuate the effects of experimentally induced stress in lab animals following both acute (Guimarães et al. 1990; Campos and Guimarães 2008; Gomes et al. 2011; Gomes et al. 2012) and chronic treatments (Campos et al. 2013). Consistent with this, other data has accumulated that CBD may also block anxiety symptoms induced by THC (Zuardi et al. 1982) or social stress under experimental conditions in healthy volunteers (Zuardi et al. 1993) or patients with social anxiety (Bergamaschi et al. 2011b). Furthermore, CBD has broadly opposite effects to that of THC, both at the behavioural and neural levels (Bhattacharyya et al. 2010; Bhattacharyya et al. 2015; Bhattacharyya et al. 2012) in healthy volunteers and evidence from double-blind randomised clinical trials point towards efficacy as an antipsychotic in patients with established psychosis (Leweke et al. 2012; McGuire et al. 2017). More recently, we have shown that a single dose of CBD may partially normalise dysfunction in the key neural substrates implicated in psychosis in CHR patients (Bhattacharyya et al. 2018). However, whether CBD could attenuate the effects of stress under experimental conditions in patients with CHR has never been tested before. This is of particular importance due to the lack of evidence of a beneficial effect of currently available antipsychotic treatments on the dysregulated neuroendocrine stress response. Therefore, the main objective of the present study was to investigate whether cannabidiol, a non-psychoactive substance present in the extract of the cannabis plant, has potential to mitigate the harmful effects of exposure to stress under experimental conditions in CHR patients.

Therefore, we investigated whether CHR patients had a blunted neuroendocrine and exaggerated anxiety response to acute exposure to social stress compared with healthy controls. Our main aim was to assess whether short-term treatment with CBD would partially normalise the altered acute neuroendocrine and anxiety response stress exposure in CHR patients. We hypothesised that relative to healthy controls, CHR patients under the influence of placebo would display the most severe alterations in the neuroendocrine and psychological responses to stress, while CBD treatment would attenuate some of these effects such that CHR patients under CBD would display an intermediate level of stress response.

## Method

### Participants

Cases consisted of 33 individuals who met the Personal Assessment and Crisis Evaluation (PACE) CHR criteria (Yung et al. 1998), recruited from a specialist clinical service for people at risk for psychosis in South London. Controls consisted of 26 age (± 3 years) and gender-matched healthy individuals, who had no history of a mental disorder, screened negative for psychotic disorder using the Psychosis Screening Questionnaire (PSQ; details below) (Bebbington and Nayani 1995) and were recruited via advertisement in local websites from the same geographical area as cases. All procedures comply with the Helsinki Declaration, as revised in 2008. Participants were reimbursed for their time and travel expenses. These procedures were approved by Psychiatry, Nursing and Midwifery Research Ethics Committee at King’s College, London (Approval number PNM/13/14-22), and NHS ethics 13/LO/0243. All participants gave written informed consent before taking part in the study and completed anonymised questionnaires in private.

### Procedure

**Fig. 1:**
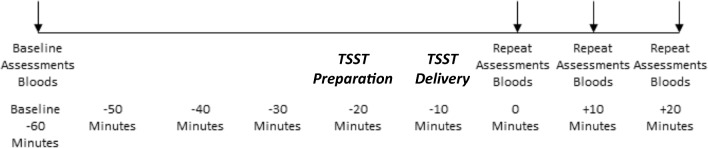
Timing of procedures for TSST

CHR participants were enrolled in a randomised, placebo-controlled, between-groups, double-blind study (Bhattacharyya et al. 2018). Sixteen participants were given oral 600 mg CBD daily and 16 received identical placebo capsules (STI Pharmaceuticals, UK). On the eighth day of the study, participants took part in the Trier Social Stress Test (TSST). Healthy participants came in for one study session and were not in any drug trial. All participants started the protocol (see Fig. 1) at approximately 10 am (− 60 min) after eating their breakfast at approximately 8.30 am. The TSST procedures commenced at approximately 11 am. Healthy participants were asked not to use cannabis for 96 h (4 days) before, alcohol for 24 h before and nicotine for 6 h before any other recreational drugs (e.g. speed, cocaine) from 2 weeks before the study. CHR participants were asked to abstain from recreational drugs throughout the trial and to not use alcohol for 24 h before the procedures and nicotine for 6 h before. All CHR participants were antipsychotic naive. All participants had a clean urine drug screen result. Participants also took part in a short health review to rule out any medical condition which may affect the endocrine system and also to ensure the healthy participants had no underlying mental health condition. Contraceptives were allowed. Participants were seated in a phlebotomy chair and a cannula was then inserted into the antecubital region of the non-dominant arm. Baseline blood samples (2 ml) were collected into serum-separating tubes. The participant then completed the baseline battery of questionnaires (State Trait Anxiety Inventory; STAI, Self-Statements during Public Speaking Scale; SSDPS).

After these had been completed (generally at 11 am; − 20-min time point), the participant took part in the TSST (see below). Following this, participants were led back to the phlebotomy chair by the experimenter. Blood samples were obtained immediately and then serial questionnaires (STAI, SSDPS) were filled out (0 min). This same battery of questionnaires and samples were then also completed at + 10 and + 20 min after completing the task. At the end of the session, participants were debriefed about the study and received reimbursement for their participation.

### Trier Social Stress Test

The Trier Social Stress Test (TSST) (Kirschbaum et al. 1993) is a well-validated stress induction paradigm that has been shown to reliably induce stress as reflected in changes in cortisol levels, under experimental conditions over the past couple of decades. This stress induction paradigm involves a social evaluative element, which make it comparable to the social stressors which individuals experience in their daily lives and arguably ecologically more valid. Participants were told they will take part in a public speaking exercise. The experimenter takes the participant into a separate room where a panel of two people were assembled and the standardised TSST instructions were read to them (see additional materials; see Fig. 1 for explanation of timing of procedures). They were then taken to a different room for the 10-min preparation period. They were informed that they would be given 10 min to prepare for a 5-min speech where they had to imagine they had an interview for their ideal job and they needed to deliver the speech to convince the panellists as to why they were ideal for that job, they were then led to an empty room to prepare. After the 10-min preparation period in a different room, participants returned to the panel to deliver their speech. Once they had completed the speech, they were informed they would take part in a mental arithmetic task as per the TSST protocol (see additional materials for details of mental arithmetic task).

### Assessment of endocrine response to stress (primary outcome)

As illustrated in Fig. 1, neuroendocrine response to stress was indexed by measuring serum cortisol level in blood samples collected at baseline (− 60 min) and at 0, +10 and + 20 min after the Trier Social Stress Test (TSST).

### Assessment of anxiety and negative thoughts about TSST in response to stress

*Anxiety* was assessed using the State Trait Anxiety Inventory (STAI) (Spielberger et al. 1970). We employed the ‘state’ subscale of the questionnaire to measure change in anxiety induced by experimental exposure to stress (STAI-S). *Reactions to public speaking* were measured using the Self-Statements during Public Speaking Scale (SSDPS) (Hofmann and DiBartolo 2000). This measures individuals’ perception of performance in relation to public speaking. It has 5 items evaluating negative thoughts and 5 evaluating positive thoughts, each rated on a 5-point Likert scale. We employed the negative subscale for this study. Both of these scales were collected at baseline (− 60 min) and at 0, + 10 and + 20 min after the Trier Social Stress Test (TSST).

### Statistical analysis

All statistical analyses were carried out using Statistical Package for Social Science (SPSS) version 22, and the outcome variables were normally distributed. *T* tests were carried out to assess whether there were group differences in sex or mean age. As there were no differences, neither were considered in the analyses. For the outcome variables of anxiety and negative self-statements, area under the curve (AUC) for the four time points was calculated by using the trapezoid formula with respect to ground, as outlined by Pruessner et al. (2003) and these were used in subsequent analyses. For cortisol, the change in cortisol level from baseline to the immediate post-TSST time point (time 0; time 0 minus baseline) was used in subsequent analyses. In accordance with our hypothesis, separate one-way analysis of variances (ANOVAs) were carried out with planned linear contrast to examine whether there was a main effect of group (HC, CHR-CBD, CHR-P) such that changes were HC > CHR-CBD > CHR-P for each of the main outcomes of interest (cortisol, anxiety and negative self-statements).

#### Missing data

Data for one item in the baseline STAI questionnaire was missing in one HC participant and for one HC participant in the baseline SSDPS-N. Similarly, data for one item in the SSDPS-N questionnaire for the 0-min time point was missing in two CHR-CBD participants and one CHR-P, and in one CHR-P participant for the STAI scale at the + 10 time point. In the case of missing values in questionnaires, an average of the scale or subscale score was estimated per participant and used in place of the missing value.

In the case of missing data for outcomes that were measured repeatedly, the last observation carried forward method was used to impute missing values. This was used in three instances of missing data in HC and once in CHR-P for the SSDPS-N and once for CHR-P for cortisol.

Some participants had incomplete data for the different time points and could not realistically be transposed and were therefore not entered into certain analyses. In the cortisol analysis, this occurred in one CHR-CBD participant. For the STAI analysis, this occurred in four healthy controls, five CHR-P participants and two CHR-CBD participants. For the SSDPS-N analysis, this occurred in three healthy controls, five CHR-P participants and two CHR-CBD participants.

## Results

All results are reported as two-tailed tests. One healthy participant was excluded from the analysis as at the time of the baseline assessment, they met criteria for possible psychotic disorder. Table 1 outlines the participants’ demographic information as well as baseline psychopathology. This outlines that CHR participants had more cannabis use than healthy controls at baseline but that cannabis use rates were the same between the Placebo and CBD groups. There was also no difference between the two CHR groups on cortisol, STAI and CAARMS positive scores but STAI was significantly higher in CHR compared with healthy control participants. Furthermore, there was no significant difference in cortisol levels between the three groups. Healthy controls had a higher level of education than both CHR groups. There was no difference in educational level between CBD and placebo groups. In the CHR-CBD group, on the day of the experiment, mean plasma CBD levels were 61.0 nM (s.d. = 45.49) about an hour before TSST commenced and 74.71 nM (s.d. = 43.65) about 40 min after TSST commenced (which was after TSST was complete).

**Table 1:** Demographics

	CHR-CBD (*n* = 16)	CHR-P (*n* = 17)	HC (*n* = 25)	*p* (HC v CHR)	*p* (HC v CHR-CBD)	*p* (HC v CHR-P)	*P* (CBD v placebo)
Gender (% female)	37.50	58.80	52	.79	.46	.63	.22
Current cannabis use (% yes)	47	41	30	.07	.24	.24	.76
Age (M, s.d.)	22.33, 5.12	25.12, 5.40	23.91, 3.93	.94	.29	.42	.14
Education level (1 HC and CHR-CBD and 2 CHR-P missing this data)	20% GCSE, 26.7% A-levels or equivalent, 40% undergraduate degree, 6.7% postgraduate degree	29.4% GCSE, 17.7% A-level or equivalent, 35.3% undergraduate degree, 5.9% postgraduate degree	24% A-levels, 44% undergraduate degree, 28% postgraduate degree	.015	.07	.02	.90
Cortisol (before drug administration; M, s.d.)	406.87, 107.02	363.82, 134.02	369.92, 162.29	.65	.44	.90	.33
STAI (before drug administration; M, s.d.)	39.57, 9.33	41.07, 8.78	32.87, 10.27	.03	.05	.02	.66
CAARMS positive symptoms score (M, s.d.)	9.88, 6.52	12.47, 8.55	NA	NA	NA	NA	.34

Table 2 outlines the descriptive statistics for each time point for all of the measures of response to stress.

**Table 2:** Descriptives for reactions to stress

	Before drug administration		Baseline		0 min		+ 10 min		+ 20 min	
Cortisol levels (nmol/L)	M	s.d.	M	s.d.	M	s.d.	M	s.d.	M	s.d.
CHR-CBD	406.87	107.02	397.15	117.9	365.67	132.94	366.54	128.16	322.71	118.11
CHR-P	363.82	134.02	343.47	121.02	297.47	115.07	298.73	137.77	274.27	125.76
Healthy control	NA	NA	369.92	162.29	447	178.34	420.72	175.83	390.8	171.31
STAI scores	M	s.d.	M	s.d.	M	s.d.	M	s.d.	M	s.d.
CHR-CBD	39.57	9.33	37.33	8.66	44.31	11.11	42.62	10.7	43	12.44
CHR-P	41.07	8.78	38.07	10.21	48.31	11.53	44	10.82	41.13	10.76
Healthy control	NA	NA	32.87	10.27	40.33	13.18	34.79	11.17	31.87	10.79
SSDPS-N scores	M	s.d.	M	s.d.	M	s.d.	M	s.d.	M	s.d.
CHR-CBD	NA	NA	9.27	3.94	10.77	3.59	10.54	3.8	10.5	5.36
CHR-P	NA	NA	10.79	4.66	13.47	5.98	13.07	6.20	11.93	5.41
Healthy control	NA	NA	8	2.88	9.42	4.74	8.14	3.27	7.73	3.47

### Cortisol reactions to TSST

**Fig. 2:**
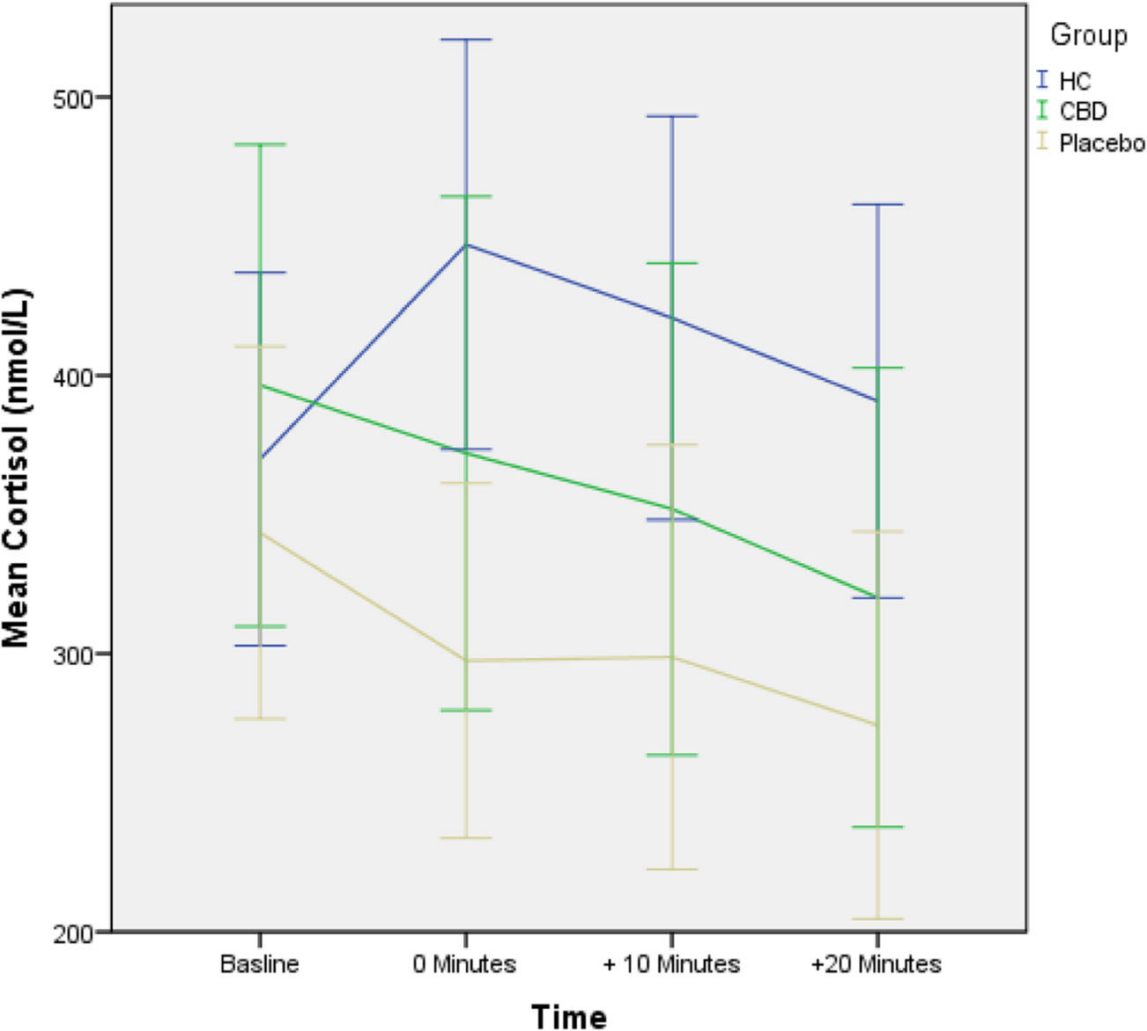
Group-dependent cortisol reaction

There was a significant effect of group (HC, CHR-P, CHR cannabidiol; CBD *F*(2,54) = 5.78, *p* = .005) on cortisol reactivity (measured as the change in the level of cortisol at 0 min relative to baseline; − 60 min). Across the three participant groups, there was a significant *F*(1,54) = 9.46, *p* = .003 linear decrease in cortisol reactivity, such that the change in cortisol (cortisol at time 0 min relative to baseline; − 60 min) associated with experimental stress exposure was greatest in HC controls and least in CHR-P patients, with CHR-CBD patients exhibiting an intermediate response (Fig. 2). Planned contrasts revealed that the cortisol reactivity was significantly different (*t*(54) = 3.08, *p* = .003; HC v CHR-P mean difference = 117.67, 95% CI 25.45–209.88, *d* = 1.31) in HC (*M* = 77.08 nmol/L, s.d. = 122.54) compared with CHR-P (*M* = − 40.59 nmol/L, s.d. = 136.98), and in HC compared with CHR-CBD (*M* = − 23.67, s.d. = 99.82; *t*(54) = 2.53, *p* = .014, mean difference = 100.75, 95% CI 4.94–196.55, *d* = 0.90) but was not different between CHR-P and CHR-CBD (*t*(54) = − .39, *p* = .70, mean difference = 16.92, 95% CI − 86.99–120.84, *d* = 0.14)).

### Anxiety reactions to acute stress induction with TSST

**Fig. 3:**
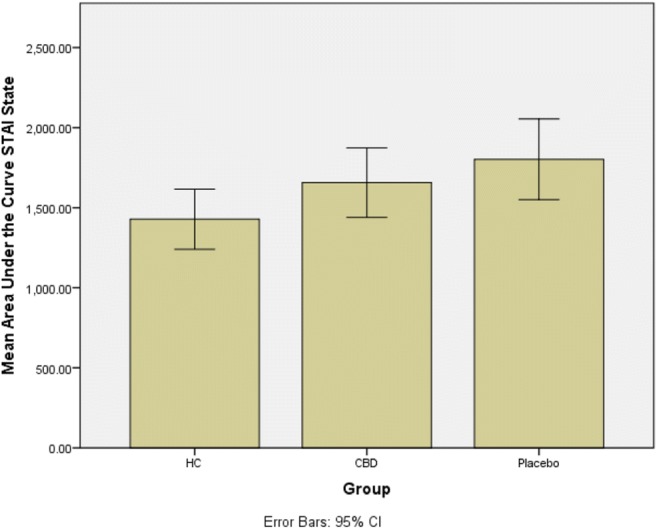
Group-dependent experience of anxiety

There was a significant effect of group (HC, CHR-P, CHR-CBD *F*(2,43) = 3.68, *p* = .034) on STAI-S AUC scores. Across the three participant groups, there was a significant (*F*(1,43) = 6.85, *p* = .012) linear increase in STAI-S scores, indicating that the experience of anxiety in response to the TSST was greatest in CHR-P and least in HC, with CHR-CBD exhibiting an intermediate response (Fig. 3). Planned contrasts revealed that the STAI-S AUC was significantly different (*t*(43) = − 2.62, *p* = .01; HC v CHR-P mean difference = − 373.75, 95% CI − 720.52–− 26.98, *d =* 0.92) in HC (*M* = 1428.33, s.d. = 413.26) compared with CHR-P(*M* = 1802.08, s.d. = 397.16), but not when compared with CHR-CBD (*M* = 1656.54, s.d. = 359.41, *t*(43) = −1.64, *p* = .11; HC v CHR-CBD mean difference = −228.21, 95% CI − 566.38–109.97, *d =* 0.59) and was also not different between CHR-P and CHR-CBD (*t*(43) = − .92, *p* = .36, mean difference = − 145.54, 95% CI − 529.16–238.07, *d =* 0.38) (see Fig. 3).

### Effect of acute stress on negative self-statements

There was a significant effect of group (HC, CHR-P, CHR-CBD *F*(2,44) = 4.57, *p* = .016) on SSDPS-N AUC scores. There was a significant *F*(1,44) = 9.11, *p* = .004 linear increase in SSDPS-N AUC scores indicating that the experience of negative statements was greatest in CHR-P, and least in HC, with CHR-CBD patients exhibiting an intermediate response. Planned contrasts revealed that the SSDPS-N was significantly different (*t*(44) = − 1.07, *p* = .004; HC v CHR-P mean difference = − 169.36, 95% CI − 309.01–− 29.71, *d* = 0.94) in HC (*M* = 333.50, s.d. = 135.82) compared with CHR-P (*M* = 502.86, s.d. = 217.12), but not when compared with CHR-CBD (*M* = 394.62, s.d. 121.58; *t*(44) = −1.12, *p* = .27; mean difference = − 61.12, 95% CI − 203.89–81.66, *d* = 0.47) and approached a significant difference between CHR-P and CHR-CBD (*t*(44) = − 1.75, *p* = .088 mean difference = 108.24, 95% CI − 46.12–262.60, *d* = 0.62).

## Discussion

The first aim of the study was to investigate whether CHR patients responded differently to a social stress paradigm compared with healthy controls. The second aim was to assess whether a 7-day treatment with CBD would attenuate the acute effects of exposure to social stress.

As expected, we found that CHR patients under placebo treatment (CHR-P) had a blunted cortisol response to the Trier Social Stress Test (TSST) compared with healthy controls (HC). Blunted cortisol response to experimental stress in CHR-P compared with HC reported here is consistent with previous reports in CHR individuals (Pruessner et al. 2013) and in those with established psychosis (Jansen et al. 1998). Our report extended these previous results in CHR participants by showing that blunted cortisol response to experimental stress was also associated with a greater psychological response in terms of anxiety and perception of public speaking as stressful in CHR-P compared with HC participants.

In line with our main hypothesis, we also found that CHR-CBD displayed intermediate levels of neuroendocrine (cortisol reactivity) and psychological [anxiety and perception of stress (negative self-statements)] response to experimental stress comparedwith CHR-P and HC. Collectively, these findings suggest that CHR participants under placebo displayed abnormal neuroendocrine and psychological responses to experimental stress compared with HC participants, and that 7-day treatment with CBD may potentially help partially attenuate these altered responses to experimental stress in CHR participants. However, one needs to be cautious in considering this interpretation, as the significant linear relationship across the 3 participant groups was mainly driven by the significant difference between HC and CHR-P on these measures. Therefore, further research is needed to investigate whether CBD may be used to influence the stress response in early psychosis. Differences in cortisol reactivity and anxiety response to stress in pairwise comparisons between CHR-P and CHR-CBD were not statistically significant, although this difference approached significance when change in perception of stress (negative self-statements) was compared between them. Future longitudinal studies in larger samples taking into account confounders are necessary to independently confirm whether CBD treatment can significantly attenuate altered responses to experimental stress in CHR participants relative to placebo treatment.

Nevertheless, our results provide preliminary evidence that CBD may affect the altered neuroendocrine as well as the psychological responses to acute stress in daily life in CHR patients. Therefore, in contrast to atypical antipsychotics, which have been shown to reduce elevated diurnal cortisol levels (Zhang et al. 2005; Cohrs et al. 2006; Mondelli et al. 2010), but not correct the blunted cortisol response observed in psychosis (Mondelli et al. 2010), CBD may potentially attenuate abnormalities in some of the main components of the acute stress response in the CHR state, future research using larger samples would be required to confirm this suggestion. These results are in line with previous research, which suggests that CBD may have anxiolytic (Bergamaschi et al. 2011b) and antipsychotic effects (Bhattacharyya et al. 2018; Leweke et al. 2012; McGuire et al. 2017).

The mechanism underlying the potential anti-stress effect of CBD is currently unclear, with multiple potential mechanisms being posited. Antipsychotic effects of CBD have been linked to its effects on levels of the endogenous cannabinoid anandamide (AEA) potentially by inhibiting its catalytic enzyme fatty acid amide hydrolase (FAAH). Recent preclinical work has also suggested that CBD may block the anxiogenic effects of chronic stress that was associated with a concomitant decrease in the expression of FAAH following CBD treatment (Fogaça et al. 2018). Anxiolytic effects of CBD were blocked by cannabinoid receptor (type 1 and type 2) antagonists but not by the serotonergic 5HT-1A receptor antagonist. However, other preclinical work suggests an effect of CBD on 5-HT_1a_ receptors (Fogaça et al. 2016; Bih et al. 2015; Russo et al. 2005) may underlie its anxiolytic effects.

To the best of our knowledge, this is the first study to have investigated the effects of short-term treatment with CBD on experimentally induced stress in the context of psychosis risk. We employed a well-validated stress induction task that has a long history (Kudielka et al. 2007a) of use for experimental stress induction. This experimental stress paradigm employed social evaluative stress, which may arguably be considered more similar to the kind of life-stress that has been linked with psychosis. While the use of a laboratory task may undermine ecological validity compared with other approaches such as experience sampling techniques, it does allow for more stringent control and standardised stress exposure. Furthermore, the task of speaking in public can be said to be life-like in that it is formed as part of a mock job interview, which is a situation that most people who experience it perceive as stressful. Further, in the healthy population sample, we recruited participants such that they were matched to the at-risk participants.

However, these strengths and the results presented herein need to be considered in light of certain caveats. In particular, we were only able to investigate a relatively modest sample size, which may have affected our ability to detect significant difference between CHR-P and CHR-CBD in pairwise comparisons as well as potential generalisability of these results. Previous studies of a similar nature have employed between 10 and 35 participants (Jansen et al. 1998; Jansen et al. 2000; van Venrooij et al. 2012). Related to this, it may be argued that the dose of CBD employed by us also affected our ability to detect an anxiolytic effect of CBD. A recent study found an anxiolytic effect of an acute dose of CBD at 300 mg but not at 100 or 900 mg (Zuardi et al. 2017), suggesting an inverted U-shaped dose response. However, a previous study (Bergamaschi et al. 2011b) also reported an anxiolytic effect during an experimental public speaking task in patients with social phobia, following a dose of 600 mg of CBD, a dose that we have employed.

Furthermore, in this study, we used venous blood sampling, which in itself can be quite stressful. However, in order to mitigate the effects of venepuncture, we used an atraumatic needle and employed an intravenous cannula that enabled us to avoid repeated venepuncture. While we cannot completely rule out the possibility that the stress of venepuncture may have added to the stress of participants, we do not believe that this would have confounded our results as such stress would have acted across all participants and only added to the social evaluative stress induced by the public speaking task, thereby contributing to how stressed participants felt overall. Furthermore, there was a gap of approximately 50 min between venepuncture and stress exposure, by which time any effects of stress from the venepuncture would have reduced substantially.

It is also important to note several additional factors may also have affected cortisol response to the Trier Social Stress Test (TSST), which were not controlled for in this study, including body mass index (Bose et al. 2009), night-shift work (Kudielka et al. 2007b), menstrual cycle and the use of contraceptives (Kirschbaum et al. 1999). However, gender distributions between the groups were not significantly different and participants were randomly allocated to the two treatment groups, making it likely that these effects would have affected the study groups to a similar extent. The cortisol levels could also have been affected by individual differences in the diurnal decline of the cortisol slope, but as the CBD and placebo group were randomised, it is reasonable to conclude that these differences would be equal between these groups. Furthermore, in a previous study (Collip et al. 2011), it has been shown that the diurnal slope did not differ between healthy controls and those at above average genetic risk for psychosis. The procedure we used for the TSST may also have been insufficient to distinguish between anticipatory and reactive cortisol. The baseline sample was taken 60 min before the onset of the stressor and the first sample after was taken 20 min after stressor onset. Previous studies have suggested that anticipatory cortisol levels may provide information when considering differences in mental health and protocols have been developed to capture this (Engert et al. 2013). Similarly, we measured cortisol levels up to 20 min after the stressful task concluded. It is agreed that cortisol levels reach a peak within 20–30 min after a stressor onset and that they return back to baseline levels within 90 min of cessation of the stressor (Fink 2000). The first sample after cessation of the stressor represents 20 min after the onset of the stressor; therefore, the + 10-min sample represents 30 min after and + 20 min represents 40 min after the onset of the stressor. It is therefore possible that the sampling is picking up anticipatory cortisol reactions and not reactive cortisol. However, the 0-min cortisol samples immediately after the TSST represents 20 min after onset of the stressor and cortisol levels in the healthy control group peak at this point and then continue to reduce, it is possible that this sample is still picking up reactive cortisol. It should also be noted that it has been found that high schizotypes have been found to have a delayed cortisol response (Walter et al. 2018). However, in this study, the peak of the cortisol response was at 15 min after the cessation of the TSST in both high and low schizotypes, and the delay was in the anticipatory rise. High schizotypes had no rise until 15 min after the cessation of the TSST whereas the low schizotypes had a rise immediately before the TSST commenced. While it is possible due to the sampling procedure, we may have missed the cortisol peak for the CHR participants, this is unlikely given the last sample is taken a full 40 min after the stressor commenced and no peak is seen in our data. Future research should follow the protocol outlined by Engert et al. (2013) and ensure adequate sampling is taken both in anticipation of the TSST and after the TSST to fully assess the increase and decrease of cortisol in response to stress. In any case, the conclusions of this study that CBD may have a potential to affect cortisol in response to stress is still a possibility.

It could also be argued that education level may affect how stressful the participants found the task, while education level was lower in CHR compared with HC, it was not different between the CBD and placebo groups, making it unlikely that our conclusions would be affect by this difference.

Notwithstanding its limitations, the present study provides a strong rationale for future studies to investigate whether CBD may have potential to mitigate the harmful effects of stress in the course of daily life by attenuating the altered neuroendocrine and psychological responses to acute stress in CHR participants.
